# The effect of decitabine-combined minimally myelosuppressive regimen bridged allo-HSCT on the outcomes of pediatric MDS from 10 years’ experience of a single center

**DOI:** 10.1186/s12887-022-03376-1

**Published:** 2022-05-27

**Authors:** Junyan Gao, Yixin Hu, Li Gao, Peifang Xiao, Jun Lu, Shaoyan Hu

**Affiliations:** 1 Department of Hematology & Oncology, Children’s Hospital of Soochow University, Suzhou, Jiangsu China; 2 Department of Pediatrics, Affiliated Hospital of Yangzhou University, Yangzhou, Jiangsu China

**Keywords:** Pediatric myelodysplastic syndrome, Allogeneic hematopoietic stem cell transplantation, Decitabine, Minimally myelosuppressive regimen, Overall survival

## Abstract

**Background:**

Myelodysplastic syndrome (MDS) is a rare disease in children and the treatment option before the allogeneic hematopoietic stem cell transplantation (allo-HSCT) is rarely reported. Our main objective was to report our single-center experience with the DNA-hypomethylating agent, decitabine-combined minimally myelosuppressive regimen (DAC + MMR) bridged allo-HSCT in children with MDS.

**Methods:**

Twenty-eight children with de novo MDS who underwent allo-HSCT between 2011 and 2020 were enrolled. Patients were divided into subgroups (refractory cytopenia of childhood [RCC] and advanced MDS [aMDS]) and treated by HSCT alone or pre-transplant combination treatment based on risk stratification. The patients’ clinical characteristics, treatment strategies and outcomes were retrospectively evaluated.

**Results:**

Twenty patients with aMDS had received pre-transplant treatment (three were treated with decitabine alone, thirteen with DAC + MMR, and four with acute myeloid leukemia type [AML-type] induction therapy). DAC + MMR was well tolerated and the most common adverse events were myelosuppression and gastrointestinal reaction. DAC + MMR had shown an improved marrow complete remission (mCR) compared with AML-type chemotherapy (13/13, 100% versus 2/4, 50%, *P* = 0.044). The median follow-up for total cohort was 53.0 months (range, 2.3-127.0 months) and the 4-year overall survival (OS) was 71.4 ± 8.5%. In the subgroup of aMDS, pretreatment of DAC + MMR resulted in a much better survival rate than AML-type chemotherapy (84.6 ± 10.0% versus 0.0 ± 0.0%, *P* < 0.001).

**Conclusions:**

The DAC + MMR bridged allo-HSCT may be recommended as a novel and effective approach.

**Supplementary Information:**

The online version contains supplementary material available at 10.1186/s12887-022-03376-1.

## Introduction

Allogeneic hematopoietic stem cell transplantation (allo-HSCT) might be the only curative approach for many children with myelodysplastic syndrome (MDS). It is routinely implemented to patients with advanced MDS (aMDS) (including refractory anemia with excessive blasts [RAEB] and RAEB in transformation [RAEB-t]) or patients with refractory cytopenia of childhood (RCC) accompanied with monosomy 7, complex karyotype, severe neutropenia or transfusion dependenc e[1, 2]. Watch-and-wait strategy or immunosuppression therapy (IST) may be a choice for RCC patients with hypocellular bone marrow (BM) and absence of monosomy 7 and complex karyotype. However, a relevant proportion of those patients still need allo-HSCT subsequently for non-response or relaps e[3]. The recent update of the European Working Group of MDS in childhood (EWOG-MDS) data showed that the survival outcomes of patients transplanted from either a human leukocyte antigen identical (HLA-identical) sibling or an unrelated donor (UD) matched for 9/10 or 10/10 HLA-loci might be almost comparabl e[4]. However, cord blood transplantation (CBT) resulted in survival rates below 30-60 %[5–7]. As for haploidentical transplantation, data is far more limited, remaining identified. Interestingly, the 5-year overall survival rate (OS) of HSCT from haploidentical family donors for pediatric patients with MDS was as high as 86% in a recent Korean cohor t[8]. Generally, there is still a paucity of data to inform the best transplant type for pediatric MDS.

As for the pre-transplant period for aMDS, a diversity of therapy strategies like intensive chemotherapy, AML-type induction chemotherapy, minimally myelosuppressive regimen (MMR) and DNA methyltransferase (DNMT) inhibitors has been investigate d[9, 10]. Intensive chemotherapy is not generally recommended due to showing no survival benefi t[11]. The acute myeloid leukemia type (AML-type) induction chemotherapy is controversial because of its somewhat severe toxicity and considerable mortalit y[10, 11]. The debut of low-dose induced remission treatment (low dose of cytarabine and anthracycline in conjunction with granulocyte colony stimulating factor [G-CSF]) was reported in 1995, being applied among aged patients with myeloid tumor and yielding certain efficac y[12]. Then, it has been continuously improved and demonstrated to be efficacious during the past decades mainly among adult MDS/AML population s[13, 14]. Intriguingly, pediatric AML patients receiving MMR (one-tenth of standard dose of cytarabine, one-half dose of anthracycline in conjunction with G-CSF) showed similar outcomes and mutation clearance levels, but significantly lower toxicity compared with those receiving standard chemotherapy in our cente r[15, 16].

Hypermethylation of critical genes was revealed in adult and childhood MDS, considered one of the disease’s driving alteration s[9, 17, 18]. In addition, hypermethylation of the promoters of various genes was associated with unfavorable prognosis in MDS, and the strategy of adopting DNA-hypomethylating agents including azacitidine (AZA) and decitabine (DAC) combination therapy is appealing for MD S[19, 20]. It has been widely recognized that low-dose DNA-hypomethylating agents could improve the quality of life and prolong survival to a significant extent for old people, especially for those unfit for allo-HSCT or intensive chemotherap y[21]. However, the role of DNA-hypomethylating agents in the treatment of childhood MDS is scarc e[22, 23].

Considering the possible advantages of disease control with good tolerability during HSCT preparation, improved antitumoral alloimmunity, reduced risk of relapse, and so on,[24–26] we have upheld a scientific hypothesis that decitabine-combined MMR strategy (DAC + MMR) bridged allo-HSCT may be a feasible way for pediatric aMDS patients with low toxicity and high efficiency. Here, we present retrospective data on the 28 children with de novo MDS who underwent allo-HSCT during the past decade in our single center. The clinical features, chemotherapy regimens, transplant characteristics, outcomes, adverse events and complications were investigated and analyzed.

## Patients and methods

### Patient population

A total of 28 pediatric MDS patients hospitalized and receiving allo-HSCT from January 2011 to December 2020 at our single center were finally enrolled in this study. Both experienced hematologists and pathologists reviewed the diagnosis of all patients. They were newly diagnosed as de novo MDS according to pediatric modification of the World Health Organization (WHO) classification. According to the current recommendations, they were categorized as RCC, RAEB and RAEB-t [27, 28]. Following the proposed categorization by *Hasle* et al., patients with RCC were termed as low-grade MDS while those with RAEB or with RAEB-t were termed as advanced MDS (aMDS) [29].

The inclusion criteria were as follows: (1) younger than 14 years of age at disease onset; (2) newly diagnosed as de novo MDS; (3) not Down syndrome (DS)-related MDS; (4) receiving allo-HSCT after diagnosis. Patients who developed AML at any time before transplantation were excluded. Cytogenetic analysis of BM cells was performed for all of the patients.

The indications for allo-HSCT among MDS patients were: (1) RCC patients with monosomy 7, 7q deletion or complex karyotype; (2) RCC patients with severe neutropenia or transfusion dependence; (3) aMDS patients.

### Ethical statement

This retrospective study was authorized by the local ethical committee of Children’s Hospital of Soochow University. The written informed consents were obtained from the patients’ parents or legal guardians. The study is carried out in accordance with the Declaration of Helsinki.

### Chemotherapy

The AML-type induction chemotherapy was similar to the protocol of AML induction remission therapy used in our cente r[15, 16]. The decitabine-combined minimally myelosuppressive regimen (DAC + MMR) included three subtypes of regimens. One subtype was “DAC + MAG”, which contained decitabine (20 mg/m^2^ once a day intravenously from the first day to the 5th day), cytarabine (10 mg/m^2^ every 12 hours subcutaneously from the 6th to 15th day), mitoxantrone (5 mg/m^2^ once a day intravenously for the 6th, 8th and 10th day) and G-CSF (5 μg/m^2^ once a day subcutaneously from the 6th to 15th day). One was aliased as “DAC + HAG”, which contained decitabine, cytarabine and G-CSF with the same usage as above and homoharringtonine (1 mg/m^2^ once a day intravenously from the 6th to 12th day). And the third one contained decitabine, cytarabine, and G-CSF with the same usage as above, and idarubicin (5 mg/m^2^ once a day intravenously from the 6th to 8th day) was abbreviated as “DAC + IDAG”. Additionally, sole decitabine treatment prior to transplantation performed as decitabine at 20 mg/m^2^ once a day intravenously for five consecutive days was applied for some aMDS patients with BM blasts slightly higher than 5%.

### Transplantation

The conditioning regimens included myeloablative conditioning (MAC) and reduced-intensity conditioning (RIC). All the regimens were busulfan and cyclophosphamide based (Bu + Cy) or fludarabine and busulfan based (Flu+Bu). The types of transplantation included HLA-identical transplantation (containing sibling donor allo-HSCT [sib-HSCT] and unrelated matched HSCT), haploidentical transplantation, and cord blood transplantation (CBT). The graft-versus-host disease (GVHD) prophylaxis contained calcineurin inhibitors (cyclosporine A or tacrolimus), mycophenolate mofetil, as well as short-term methotrexate.

### Evaluation and criterion

The neutropenia was defined as absolute neutrophil count (ANC) < 1.5*10^9^ /L and severe neutropenia was ANC < 0.5*10^9^ /L. The thrombocytopenia was defined as platelet count (Plt) < 100*10^9^ /L, and severe thrombocytopenia was Plt < 20*10^9^ /L and/or clinical need for platelet transfusion. The response to treatment was assessed by reference to the International Working Group (IWG) response criteria in myelodysplasia [30]. Marrow complete remission (mCR) referred to the achievement of marrow blasts ≤5% with or without improved cytopenias. Adverse events of administered treatments were graded by using the common terminology criteria of adverse events score (CTCAE) (version 4.0). Graft failure (the primary) was defined as ANC that did not maintain sustained engraftment (> 0.5*10^9^ /L) within 28 days post-transplantation. The granulocyte engraftment was defined as ANC ≥0.5*10^9^/L for three consecutive days. The platelet engraftment was defined as Plt ≥20*10^9^/L for seven consecutive days without platelet transfusion support. The acute and chronic graft-versus-host disease (GVHD) were graded based on traditional criteria [31, 32].

### Follow-up

All the patients were followed up every month and the follow-up endpoint was August 31, 2021. The overall survival time (OS) was calculated from the date of first diagnosis to the date of death or last follow-up. The events included death, relapse, graft failure, severe complications (acute renal failure, for instance) and secondary tumor (progression to AML, for instance) and the event-free survival time (EFS) was defined as survival without those events. Relapse was defined as morphological evidence of disease in BM or recurrence and sustained pre-transplant chromosomal abnormalities. The relapse-free survival (RFS) time was calculated.

### Statistical analysis

The continuous variables with normal distribution were expressed as mean and standard deviation, while variables with skewed distribution were expressed as median and range. The categorical variables were described as number and percentage. The independent-samples T test was used to assess normal distributional variables. The Mann-Whitney U test and Kruskal-Wallis H test were used to assess skewed distributional variables, as appropriate. The categorical variables were analyzed using Chi square or Fisher’s Exact Test, as appropriate. The Kaplan-Meier methods were used to describe survival functions and the log-rank test was used to compare the survival curves. A Cox’s proportional hazards regression model was used to determine the significance of risk factors for the outcomes. Factors with at least *P*-value< 0.10 in the univariate analysis were included in the model. Hazard ratios (HR) and 95% confidence intervals (CI) were calculated. SPSS 26.0 software was employed for data processing. GraphPad Prism 8.0.2 software was served as the tool for results visualization. Two-tailed *P*-values< 0.05 were considered statistically significant.

## Results

### Patients’ general features

From 2011 to 2020, 28 children with de novo MDS receiving HSCT met inclusion criteria. The general features of the 28 patients were shown in Table 1. The median age at diagnosis was 79.5 months (range, 19-138 months). Diagnosis were low-grade MDS (RCC, *n* = 7) and aMDS (RAEB, *n* = 15 and RAEB-t, *n* = 6). At diagnosis, 89.3% (25/28) of patients had cytopenia involving at least two lineages and 32.1% (9/28) had severe neutropenia (Table 2). The chromosome abnormalities accounted for 35.7% (10/28), and mainly were monosomy 7 (*n* = 6), trisomy 8 (*n* = 2), complex karyotype (*n* = 1), and + 1, der (1;12)(q10;q10) (*n* = 1) (Table 2). Of the 21 aMDS patients, 20 patients were treated pre-HSCT, while only one patient went directly to HSCT (patient 11, Table 2). Among treated patients, 65.0% (13/20) received DAC + MMR, 15.0% (3/20) had sole decitabine, and 20.0% (4/20) accepted AML-type chemotherapy. The median age at HSCT was 81.5 months (range, 21-152 months). The majority of patients underwent myeloablative conditioning (23/28, 82.1%). Transplantation was performed between 2011 and 2015 in 11 (11/28, 39.3%) patients and between 2016 and 2020 in 17 (17/28, 60.7%) patients. Transplant types were HLA-identical HSCT in 7 cases, haploidentical HSCT in 18 cases and CBT in 3 cases. Until August 31, 2021, none of the patients lost follow-up and none of the survivals relapsed.

**Table 1:** Baseline features, treatments and overall outcomes of the 28 children with de novo MDS

Features	Number of patients (%)
Gender	
Male	17 (60.7%)
Female	11 (39.3%)
Age at diagnosis (months)	
Median	79.5
Range	19-138
MDS subtypes	
Initial subtype: RCC	7 (25.0%)
Advanced subtypes:	21 (75.0%)
RAEB	15 (53.6%)
RAEB-t	6 (21.4%)
Karyotypes	
Normal	18 (64.3%)
Abnormal	10 (35.7%)
Monosomy 7	6 (21.4%)
Trisomy 8	2 (7.1%)
Complex karyotype	1 (3.6%)
Other	1 (3.6%)
Chemotherapy prior to HSCT	
None	8 (28.6%)
AML-type induction	4 (14.3%)
Decitabine alone	3 (10.7%)
DAC + MMR	13 (46.4%)
Age at transplantation (months)	
Median	81.5
Range	21-152
Conditioning regimen	
MAC	23 (82.1%)
RIC	5 (17.9%)
Conditioning regimens	
Bu/Cy-based	10 (35.7%)
Flu/Bu-based	18 (64.3%)
Transplant types	
HLA-identical HSCT	7 (25.0%)
Haploidentical HSCT	18 (64.3%)
CBT	3 (10.7%)
Transplant year	
2011-2015	11 (39.3%)
2016-2020	17 (60.7%)
Follow-up time (months)	
Median	53.0
Range	2.3-127.0
Time after transplantation (months)	
Median	50.2
Range	0-120.4
Graft failure	
Yes	2 (7.1%)
No	26 (92.9%)
Relapse	
Yes	0 (0%)
No	28 (100%)
Death	
Yes	8 (28.6%)
No	20 (71.4%)

*MDS* Myelodysplastic syndrome, *RCC* Refractory cytopenia of childhood, *RAEB* Refractory anemia with excessive blasts, *RAEB-t* RAEB in transformation, *HSCT* Hematopoietic stem cell transplantation, *AML* Acute myeloid leukemia, *DAC + MMR* Decitabine combined with minimally myelosuppressive regimen, *MAC* Myeloablative conditioning, *RIC* Reduced-intensity conditioning, *Bu* Busulfan, *Cy* Cyclophosphamide, *Flu* Fludarabine, *CBT* Cord blood transplantation

**Table 2:** Chemotherapy characteristics and responses of the 28 children with de novo MDS

Patient No.	MDS subtypes	Age/Gender	Cytogenetics	Cytopenias	BM blasts at diagnosis (%)	Chemotherapy regimens	Cycles	BM blasts prior to HSCT (%)	Diagnosis to HSCT (months)	Survival status	Follow-up (months)
1	RAEB-t	130/F	−7	A	22.0	DAC + MAG	2	0.0	3.83	Alive in remission	49.5
2	RAEB-t	20/M	Normal	T/N	22.5	DAC + IDAG	3	0.5	7.7	Alive in remission	31.0
3	RAEB-t	37/M	Normal	T	20.0	DAC + MAG	1	0.0	1.4	Alive in remission	43.9
4	RAEB-t	42/M	Normal	A/N,SN	20.0	DAC + MAG	2	0.0	4.47	Alive in remission	38.9
5	RAEB-t	102/M	Normal	A/T/N,SN	23.5	DAC + MAG	1	1.0	2.37	Died of aGVHD (grade 4) and TMA	5.7
6	RAEB-t	68/F	Normal	A/T/N	21.0	DAC + MAG	2	4.0	3.26	Alive in remission	105.5
7	RAEB	86/M	Normal	A/T/N	17.0	AML-type induction	2	6.0	2.85	Died of aGVHD (grade 4) and severe lung infection	6.9
8	RAEB	52/M	Normal	A/T/N,SN	16.0	DAC + MAG	2	1.0	2.53	Alive in remission	70.6
9	RAEB	110/F	Normal	A/T/N	15.0	AML-type induction	2	3.0	4.36	Died of severe lung infection	10.2
10	RAEB	72/M	Normal	A/T/N,SN	18.0	DAC + MAG	1	4.0	2.68	Alive in remission	106.3
11	RAEB	130/F	−7	A/T/N	6.0	None	0	6.0	2.3	Alive in remission	61.3
12	RAEB	69/M	−7	A/N	8.0	DAC + MAG	2	4.0	2.27	Died of aGVHD (grade 4) and severe lung infection	7.8
13	RAEB	27/M	-7	A/T/N	15.0	AML-type induction	4	3.0	6.9	Died of severe lung infection	14.7
14	RAEB	84/M	Normal	A	6.0	DAC + IDAG	2	0.5	3.23	Alive in remission	28.2
15	RAEB	72/F	+ 1, der(1;12)(q10;q10)	A/T	6.0	DAC + HAG	3	1.0	3.4	Alive in remission	23.1
16	RAEB	19/M	+ 8,+ 9,-19,+ 20	A/T/N,SN	18.0	DAC + MAG	1	0.0	1.73	Alive in remission	56.5
17	RAEB	136/M	+ 8	A/T/N,SN	14.0	AML-type induction	2	8.5	4.07	Graft failure; died of disease progression	5.4
18	RAEB	56/F	Normal	A/T/N	6.0	DAC + HAG	1	0.0	6.63	Alive in remission	75.2
19	RAEB	109/M	Normal	A/T/N	6.0	DAC alone	1	0.0	2.43	Alive in remission	73.5
20	RAEB	37/F	Normal	A/T/N	5.5	DAC alone	1	0.0	6.57	Alive in remission	127.0
21	RAEB	96/M	+ 8	A/T/N	6.0	DAC alone	2	0.0	3.2	Died of aGVHD (grade 4) and MSOF	3.5
22	RCC	74/F	-7	A/T	2.0	None	0	2.0	2.2	Alive in remission	81.9
23	RCC	77/M	-7	A/T/N,SN	3.0	None	0	0.0	2.27	Suffered sudden cardiac death on day 1 after transplantation	2.3
24	RCC	104/F	Normal	A/T/N,SN	4.0	None	0	1.0	3.87	Alive in remission	66.1
25	RCC	129/M	Normal	A/T/N,SN	4.0	None	0	0.0	1.17	Alive in remission	61.0
26	RCC	82/M	Normal	A/T/N	3.0	None	0	1.0	2.33	Alive in remission	59.6
27	RCC	112/F	Normal	A/T/N	2.0	None	0	0.0	1.8	Alive in remission	72.0
28	RCC	138/F	Normal	A/T/N	3.0	None	0	0.0	2.03	Alive in remission	95.8

*MDS* Myelodysplastic syndrome, *BM* Bone marrow, *HSCT* Hematopoietic stem cell transplantation, *RCC* Refractory cytopenia of childhood, *RAEB* Refractory anemia with excessive blasts, *RAEB-t* RAEB in transformation, *A* Anemia, *T* Thrombocytopenia, *N* Neutropenia, *SN* Severe neutropenia, *DAC* Decitebine, *MAG* Mitoxantrone, cytarabine and G-CSF, *IDAG* idarubicin, cytarabine and G-CSF, *HAG* Homoharringtonine, cytarabine and G-CSF, *aGVHD* Acute graft-versus-host disease, *TMA* Thrombotic microangiopathy, *MSOF* Multiple system organ failure

### Response to chemotherapy

The pre-transplant treatments and responses of each patient were summarized in Table 2. During the pre-transplant period, different strategies were applied to the patients according to the attending’s decision and patient’s agreement.

Three aMDS patients with BM blasts slightly higher than 5% were treated with sole decitabine prior to transplantation. One patient received two cycles of decitabine and achieved mCR. One patient achieved mCR after one cycle of decitabine. The rest one patient was a 3-years old girl at diagnosis, and traditional Chinese medicine was taken without medical advice since December 2011 (patient 20, Table 2). One cycle of decitabine and subsequent allo-HSCT were performed in 2015, and she had achieved mCR before transplantation. Thirteen aMDS patients (seven were RAEB and six were RAEB-t) received DAC + MMR and a total of 23 cycles of DAC + MMR were administered. All of them achieved mCR before transplantation. Two of the four patients who received AML-type induction therapy achieved mCR, while the other two gained 6.0 and 8.5% of BM blasts before transplantation (Table 2). Eight patients (including seven RCC patients and one aMDS patient [patient 11, Table 2]) proceed to transplantation directly.

### Adverse events on decitabine concomitant chemotherapy

A total of 27 cycles of decitabine were administered among the 16 patients, of which, 4 cycles were sole decitabine therapy for three patients and 23 cycles were decitabine-combined MMR for 13 patients.

The most common hematologic toxicity was myelosuppression. At the beginning of decitabine-concomitant treatment, 12 of 16 patients (75.0%) had neutropenia, and 5 of the 16 patients (31.3%) had severe neutropenia. During the decitabine-concomitant therapy, four patients with previously normal ANC developed neutropenia (*n* = 2) or severe neutropenia (*n* = 2), and five patients with initial neutropenia worsen to severe neutropenia. 14 of the 27 cycles (51.9%) involved neutropenia, and 13 of the 27 cycles (48.1%) involved severe neutropenia. Twelve patients had severe thrombocytopenia during the treatment, involving 13 of the 27 cycles (48.1%). The non-hematologic toxicities were mainly mild and the most common complications were gastrointestinal problems and infection. Nine episodes of infection were observed in eight patients and eight of the nine episodes occurred in severe neutropenia. There was no delay or reduction during the sole decitabine therapy for the three patients. As for decitabine-combined MMR therapy, treatment was delayed in two cycles. Vomiting (CTCAE grade 3) and gastritis led to prolonged treatment after one cycle of “DAC + IDAG” in one patient (patient 14, Table 2). Acute pancreatitis led to treatment discontinuation after two cycles of “DAC + MAG” in one patient (patient 1, Table 2).

### Allogeneic hematopoietic stem cell transplantation

All the 28 MDS patients enrolled in the present study proceeded to allo-HSCT, and depending on the transplant types, they were divided into three groups (HLA-identical HSCT treated group [*n* = 7], haploidentical HSCT treated group [*n* = 18] and CBT treated group [*n* = 3]). There were no statistical differences in the age at transplantation, time interval from diagnosis to transplantation, gender distribution, gender and blood type of donor and recipient compatibility, conditioning regimen among the three groups (Table 3). Compared with patients with haploidentical HSCT, patients with HLA-identical HSCT accessed younger donors (*P <* 0.001) (Table 3). Patients with CBT were transfused significantly fewer mononuclear cells and CD34+ cells when compared with patients in other two groups (both *P* = 0.023) (Table 3). There were 7 (7/7, 100%), 18 (18/18, 100%) and 1 (1/3, 33.3%) patients who were successfully engrafted in granulocyte among HLA-identical HSCT group, haploidentical HSCT group and CBT group, respectively. The median time for granulocytic engraftment were 13.0 (11.0-20.0), 14.0 (10.0-18.0), and 19.0 (19.0-19.0) days, respectively. There were 7 (7/7, 100%), 17 (17/18, 94.4%) and 1 (1/3, 33.3%) patients reached platelet engraftment among the three groups and the median time were 14.0 (11.0-40.0), 15.0 (9.0-66.0) and 22.0 (22.0-22.0) days, respectively. There were no statistical differences in the incidence of the main complications (aGVHD, cGVHD, cytomegalovirus infection, Epstein-Barr virus infection, other infections, bronchiolitis obliterans, and so on) (Table 3).

**Table 3:** Transplant characteristics and outcomes of the 28 children with de novo MDS

Characteristics	HLA-identical HSCT (*n* = 7)	Haploidentical HSCT (*n* = 18)	Cord blood transplantation (*n* = 3)	*P*-value
Recipient age (months)				0.989
Median	87	80	79	
Range	44-132	21-152	34-140	
Recipient gender, *N*				0.747
Male	5	10	2	
Female	2	8	1	
Donor age (months)			–	< 0.001
Median	151	376	–	
Range	31-321	288-468	–	
Gender match between recepient and donor, *N*				0.772
Match	4	9	–	
Male to female	1	5	–	
Female to male	2	4	–	
Blood type compatibility, *N*				0.279
Compatible	2	11	–	
Minor incompatible	3	3	–	
Major incompatible	2	4	–	
Conditioning regimen, *N*				0.544
MAC	5	15	3	
RIC	2	3	0	
Conditioning regimens, *N*				0.211
Bu/Cy-based	4	6	0	
Flu/Bu-based	3	12	3	
Total number of mononuclear cells (10^8^/kg)				0.023
Median	9.48	9.66	1.90	
Range	3.84-11.10	2.17-32.53	1.68-3.40	
Total number of CD34+ cells (10^6^/kg)				0.023
Median	5.15	5.47	0.70	
Range	1.69-10.70	1.23-16.68	0.62-1.60	
Granulocytic engraftment (days)				0.295
vMedian	13.0	14.0	19.0 ^a^	
Range	11.0-20.0	10.0-18.0	19.0-19.0 ^a^	
Megakaryocytic engraftment (days)				0.615
Median	14.0	15.0 ^b^	22.0 ^a^	
Range	11.0-40.0	9.0-66.0 ^b^	22.0-22.0 ^a^	
GVHD prophylaxis				0.156
CsA/MMF	1	8	2	
CsA/MMF/MTX	5	7	0	
FK/MMF	1	0	0	
FK/MMF/MTX	0	3	1	
Acute GVHD, *N*	2	11	2	0.357
Grade of aGVHD				0.499
None	5	8	1	
Grade I-II	2	7	2	
Grade III-IV	0	3	0	
Chronic GVHD, *N*	0	3	0	0.393
Grade of cGVHD				0.393
None	7	15	3	
Limited	0	3	0	
Extensive	0	0	0	
CMV infection, *N*	2	7	1	0.886
EBV infection, *N*	1	1	0	0.658
Other infections, *N*	5	9	1	0.476
Bronchiolitis obliterans, *N*	2	1	0	0.202
Graft failure, *N*	0	0	2	< 0.001
Death, *N*	0	5	3	0.006
Relapse, *N*	0	0	0	1.000
4-year OS	100.0 ± 0.0%	72.2 ± 10.6%	0.0 ± 0.0%	< 0.001
4-year EFS	100.0 ± 0.0%	66.7 ± 11.1%	0.0 ± 0.0%	0.001
Follow-up (months)				0.043
Median	61.3	53.0	5.4	
Range	28.2-127.0	3.5-106.3	2.3-14.7	

^a^Only one patient achieved neutrophil and platelet engraftment among the three patients with cord blood transplantation

^b^One of the eighteen patients with haploidentical HSCT didn’t achieve platelet engraftment

*MDS* Myelodysplastic syndrome, *HSCT* Hematopoietic stem cell transplantation, *MAC* myeloablative conditioning, *RIC* Reduced-intensity conditioning, *Bu* Busulfan, *Cy* Cyclophosphamide, *Flu* Fludarabine, *GVHD* Graft-versus-host disease, *CsA* Cyclosporin a, *MMF* mycophenolate mofetil, *MTX* Methotrexate, *FK* Tacrolimus, *CMV* Cytomegalovirus, *EBV* Epstein-Barr virus, *OS* Overall survival, *EFS* Event-free survival

### Survival and main complications

The median follow-up within the whole cohort was 53.0 months (range, 2.3-127.0 months), while the median follow-up of the survivors (*n* = 20) was 63.7 months (range, 23.1-127.0 months). By August 31, 2021, 20 patients (20/28, 71.4%) were alive without evidence of MDS (Table 2). The causes of death of 8 patients were reviewed and listed in Table 2. Four patients died of severe aGVHD. Two patients died of severe lung infection. One patient with CBT presented with graft failure and died 2 months after transplantation due to disease progression. One with CBT suffered sudden cardiac death on day one after transplantation.

**Fig. 1:**
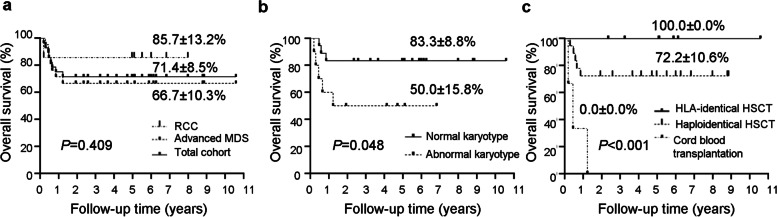
The overall survival of the whole 28 de novo MDS patients with allo-HSCT. **a** The overall survival of RCC patients (*n* = 7) compared with aMDS patients (*n* = 21). **b** The overall survival of patients with normal karyotype (*n* = 18) compared with those with abnormal karyotypes (*n* = 10). **c** The overall survival of patients with different transplant types. MDS: myelodysplastic syndrome; allo-HSCT: allogeneic hematopoietic stem cell transplantation; RCC: refractory cytopenia of childhood; aMDS: advanced myelodysplastic syndrome

The 4-year OS rate for the total cohort was 71.4 ± 8.5%, while that for patients with RCC and those with aMDS was 85.7 ± 13.2% and 66.7 ± 10.3%, respectively (Fig. 1a). Patients with abnormal karyotypes at diagnosis had significantly low survival rate when comparing with patients whose karyotypes at diagnosis were normal (50.0 ± 15.8% versus 83.3 ± 8.8%, *P* = 0.048) (Fig. 1b). Compared with transplantation from HLA-identical (matched sibling or unrelated) donors for 9/10 or 10/10 HLA-loci (4-year OS, 100.0 ± 0.0%), the outcomes of haploidentical HSCT were also satisfactory with an estimated 4-year OS of 72.2 ± 10.6%, while the three patients with CBT showed a dismal survival (0.0 ± 0.0%) (Fig. 1c).

**Fig. 2:**
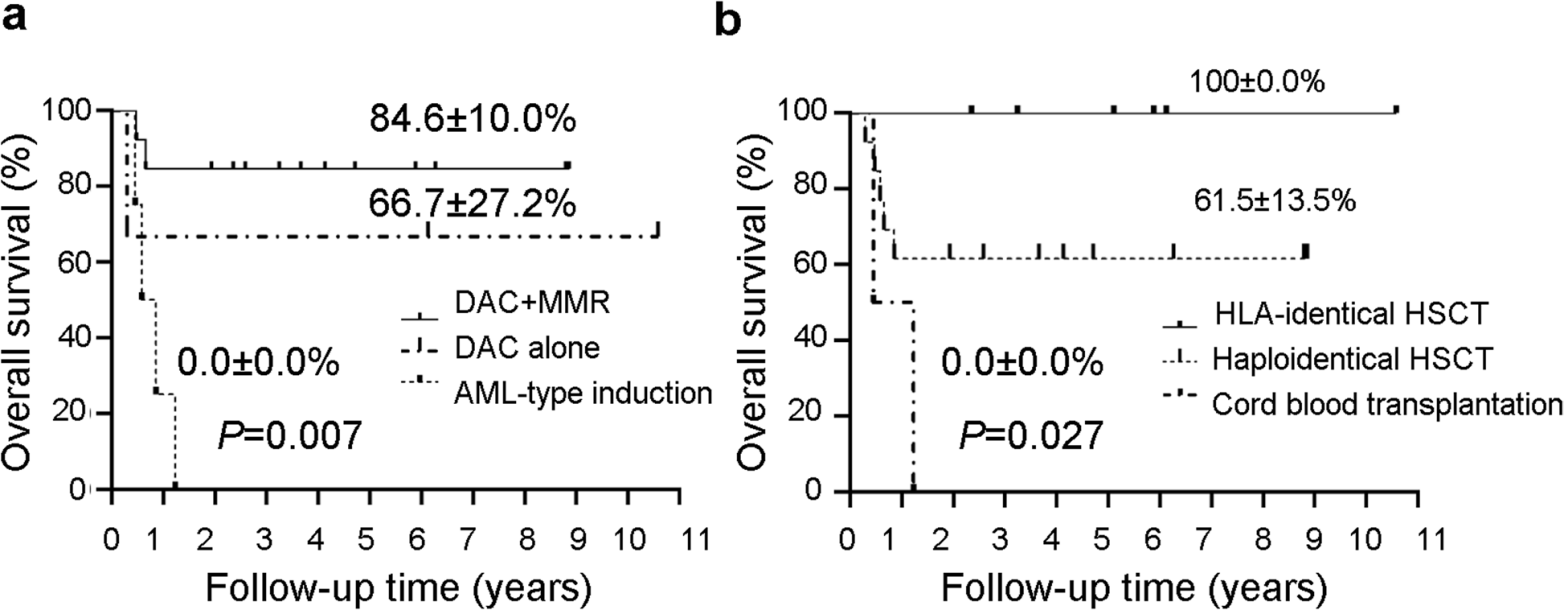
The overall survival of the subgroup of the 21 aMDS patients with allo-HSCT. **a** The overall survival of aMDS patients with different bridging treatment strategies. **b** The overall survival of aMDS patients with different transplant types. aMDS: advanced myelodysplastic syndrome; allo-HSCT: allogeneic hematopoietic stem cell transplantation

Based on the different bridging treatment strategies, the 21 aMDS patients were further analyzed (Fig. 2). One aMDS patient with 6% BM blasts underwent HSCT directly and has been alive without MDS. With respect to the 20 aMDS patients with pretreatment, the rate of the subgroup of the 13 patients treated with DAC + MMR was as high as 84.6 ± 10.0%. The three patients with DAC alone came out an estimate 4-year OS of 66.7 ± 27.2%. At the same time, all of the four patients treated with AML-type induction therapy followed by HSCT died soon after transplantation (Fig. 2a). Among the 13 aMDS patients with haploidentical HSCT, five patients died, and four of the five ones died of severe aGVHD, and it finally showed a 4-year OS of 61.5 ± 13.5% (Fig. 2b).

In addition, potential risk factors including gender, age at diagnosis, cytogenetics at diagnosis, age at transplantation, time interval from diagnosis to transplantation, percentage of BM blasts at diagnosis or before transplantation, conditioning regimen, transplant type, transplantation period and acute/chronic GVHD were analyzed. The factors with *P* < 0.10 (the factors included cytogenetics at diagnosis [*P* = 0.066], percentage of BM blasts at transplantation [*P* = 0.024], transplant type [*P* = 0.001] and acute GVHD [*P* = 0.001] for the whole MDS cohort, while the factors were percentage of BM blasts at transplantation [*P* = 0.028], transplant type [*P* = 0.013] and acute GVHD [*P* = 0.005] for the aMDS patients) in univariate analysis were further taken into the multivariate analysis (Tables 4 and 5). Finally, CBT (HR = 49.272, 95% CI 2.868-846.433, *P* = 0.007 for the whole cohort, HR = 7.973, 95% CI 1.180-53.882, *P* = 0.033 for the subgroup of aMDS patients) and grade III-IV of aGVHD (HR = 64.283, 95% CI 4.605-897.352, *P* = 0.002 for the whole cohort, HR = 14.757, 95% CI 1.556-139.928, *P* = 0.019 for the subgroup of aMDS patients) were demonstrated to be the independent prognostic factors for OS (Table 5).

**Table 4:** The univariate and multivariate analyses of the risk factors for OS among the 28 patients with de novo MDS

Variables	Univariate analysis			Multivariate analysis		
	HR	95% CI	*P*-value	HR	95% CI	*P*-value
Age at diagnosis	1.007	0.988-1.027	0.477			
Diagnosis, advanced MDS	2.356	0.289-19.172	0.423			
Gender, male	2.435	0.490-12.100	0.277			
Cytogenetics at diagnosis, abnormal karyotype	3.834	0.914-16.082	0.066	0.728	0.108-4.935	0.745
Age at transplantation	1.007	0.988-1.026	0.504			
Time interval from diagnosis to HSCT	1.038	0.713-1.509	0.847			
BM blast prior to HSCT	1.361	1.040-1.779	0.024	0.964	0.664-1.398	0.846
Conditioning regimen, MAC	28.852	0.018-45,615.670	0.371			
Conditioning regimens, Flu/Bu	1.781	0.359-8.832	0.480			
Transplantation type, CBT	8.294	2.275-30.237	0.001	49.272	2.868-846.433	0.007
Transplantation period, early period (2011-2015)	0.753	0.152-3.736	0.728			
Acute GVHD, grade III-IV	18.137	3.177-103.523	0.001	64.283	4.605-897.352	0.002

*OS* Overall survival, *MDS* Myelodysplastic syndrome, *HSCT* Hematopoietic stem cell transplantation, *BM* Bone marrow, *MAC* Myeloablative conditioning, *Bu* Busulfan, *Flu* Fludarabine, *CBT* Cord blood transplantation, *GVHD* Graft-versus-host disease

**Table 5:** The univariate and multivariate analyses of the risk factors for OS among the 21 patients with advanced MDS

Variables	Univariate analysis			Multivariate analysis		
	HR	95% CI	*P*-value	HR	95% CI	*P*-value
Age at diagnosis	1.016	0.995-1.038	0.134			
Gender, male	1.563	0.302-8.085	0.594			
Cytogenetics at diagnosis, abnormal karyotype	2.596	0.580-11.618	0.212			
BM blast prior to HSCT	1.398	1.036-1.886	0.028	1.100	0.708-1.708	0.672
Age at transplantation	1.017	0.995-1.038	0.131			
Time interval from diagnosis to HSCT	0.969	0.645-1.456	0.878			
Conditioning regimens, Flu/Bu	1.589	0.308-8.199	0.580			
Transplantation type, CBT	5.165	1.423-18.752	0.013	7.973	1.180-53.882	0.033
Transplantation period, early period (2011-2015)	1.561	0.349-6.984	0.560			
Acute GVHD, grade III-IV	13.599	2.222-83.238	0.005	14.757	1.556-139.928	0.019

*OS* Overall survival, *MDS* Myelodysplastic syndrome, *HSCT* Hematopoietic stem cell transplantation, *BM* Bone marrow, *MAC* Myeloablative conditioning, *Bu* Busulfan, *Flu* Fludarabine, *CBT* Cord blood transplantation, *GVHD* Graft-versus-host disease

## Discussion

Here, we reported a cohort of children with MDS who underwent allo-HSCT over the past decade at our single center. To the best of our knowledge, this may be the latest research in China for systematically reviewing a certain size of cohort regarding pediatric MDS with transplantation and is also the first domestic study in China that reported the experience of decitabine-combined minimally myelosuppressive regimen prior to allo-HSCT for pediatric aMDS.

Pediatric MDS is a heterogeneous group of clonal disorder accounting for less than 5% of childhood hematological malignancies. The morphology, cytogenetics and therapy approaches would profoundly influence the survival outcome s[33]. It is recognized that patients with abnormal karyotype such as monosomy 7 or complex karyotype are more likely to progress to advanced disease and have poor outcome s[34, 35]. In the present study, ten patients with abnormal karyotypes had a significant low survival rate compared to 18 patients whose karyotypes were normal. However, the cytogenetic data is far more limited with great heterogeneity which should be carefully interpreted. Recently, with the increased access to gene mutation landscape, genetic counseling for both patients and their families would affect pediatric MDS’s clinical diagnosis and therapeutic decision s[36–38]. The gene mutation assay was performed among the 16 patients of this cohort and 14 of them were verified to carry different gene mutations (Supplementary Table 1). It will be a great challenge for pediatric hematologists further to explore the underlying conditions and their hematopoietic impacts.

As for treatment strategy, it is widely accepted that allogeneic HSCT is the only curative treatment for pediatric MD S[5, 39]. Especially, high-risk subtype of MDS is recommended to receive an early transplantation. Allo-HSCT for pediatric MDS has been adeptly mastered during the past decade in our center. The 4-year OS as high as 71.4 ± 8.5% for the total cohort, 85.7 ± 13.2% and 66.7 ± 10.3% for low-grade and advanced MDS respectively are revealed in our study, consistent with that of recent reports ranging from 30 to 80 %[1, 5, 40, 41]. It is reported that allo-HSCT from a matched related or unrelated donor offers a superior survival probability for pediatric MD S[4]. The data of the seven patients with HLA-identical transplantation in our center confirmed this conclusion again. Our preliminary data showed that the 4-year OS of haploidentical HSCT was 72.2 ± 10.6%, which indicated that haploidentical HSCT would be a feasible alternative among childhood MDS for an urgent need of transplantation. Consistently, a Korean group reviewed 36 pediatric patients with MDS who proceeded to haploidentical HSCT (*n* = 9) or HLA-identical HSCT (*n* = 24) or CBT (*n* = 3 )[8]. Intriguingly, the OS of HSCT from haploidentical family donors was comparable with that from HLA-identical donors (86% versus 79%, *P* = 0.625 )[8]. With the theoretic advantages, including low incidence of acute and chronic GVHD, despite multiple HLA mismatching and so on, cord blood has been considered as an attractive source for transplantatio n[42, 43]. However, in our center, all the three patients with CBT in the cohort died soon after transplantation, leading to no obvious benefit regarding overall survival. In the future, more data of CBT will be needed to draw certain conclusions. The cumulative incidence of transplantation-related mortality (TRM) for the total cohort was 28.6 ± 8.5%. Acute GVHD is a serious transplant complication that contributes TRM after allo-HSC T[43]. In the risk factor analysis for OS, grade III-IV aGVHD was associated with higher risk of mortality and should be prevented.

It is challenging and time-consuming for donor searching and HSCT preparation. Therefore, the disease should be controlled through a bridging treatment based on risk stratification. What has been reached as a common consensus is that conventional chemotherapy is dubious, especially for high-risk MDS. The advent of epigenetic treatment options for myeloid disorders has led to the combination concepts, and their integration with transplantation already shows a reliably improved outcome in adult MD S[24, 44]. However, the experience among pediatric MDS is far more limited with anecdotal report s[22, 45]. In our study, an excellent response rate of 100% (100.0% achieved mCR) was observed using decitabine-combined MMR with a median of two cycles (range, 1-3) for pediatric advanced MDS. At the same time, three patients with RAEB achieved mCR after one or two cycles of sole decitabine. More encouragingly, the strategy of low-dose decitabine-combined-MMR use proved to be very tolerable with mild non-hematologic toxicity in the pediatric population. Considering the heterogeneity of MDS and unevenly distributed subgroups, patients with advanced MDS were further extracted and analyzed to better illustrate the effect of decitabine-combined therapy bridged allo-HSCT. As a result, 13 patients with DAC + MMR treatment showed a quite inspiring survival (84.6 ± 10.0%), and none of the 11 survivals relapsed at last follow-up. DAC + MMR appears to be a promising bridge to HSCT with its high efficiency of eliminating the excess BM blasts with low toxicity. These exciting results provided a valuable clinical experience for the use of decitabine in the pediatric population.

Preemptive treatment for the minimal residual disease (MRD) is essential for preventing or substantially delaying hematological relapse after HSCT in pediatric MDS, especially in high-risk subgroups. The discovery of genome-wide DNA hypermethylation in pediatric MDS provides a rationale for DNMT inhibitors applicatio n[9, 17]. Low-dose decitabine could directly and irreversibly inhibit the DNA methyltransferases. More intriguing potential of decitabine among antitumoral alloimmunity and pro-apoptotic effect of tumor cells has emerged in recent year s[24–26, 45]. The MMR is originated from the low-dose chemotherapy consisting of low-dose cytarabine and aclarubicin combined with G-CSF, abbreviated as “CAG”, proposed in 1995. The CAG regimen achieved certain efficacy in refractory/relapse adult MDS and AM L[12]. Even in low/intermediate risk adult MDS and AML, the CR rates of low-dose induction therapy were significantly higher than intensive chemotherap y[46]. However, the cardiac toxicity associated with aclarubicin mainly limited to a certain extent of the application of CAG regimen. Then, alternatives with similar therapeutic effect and mild cardiac toxicity were developed, forming different regimens of MMR. The advantages of MMR may be due to the synergy of G-CSF and low-dose chemotherapy drugs. G-CSF priming could preferentially potentiate Ara-C and anthracycline-mediated cytotoxicity on myeloid tumor cells, presumably by enhancing G0 resting tumor cells into the cell cycl e[47]. In addition, the G-CSF combination may inhibit the self-renewal capacity of myeloid tumor cells and leukemia stem cell s[48, 49]. It will be of great interest to investigate the underline specific mechanisms in the future. Hence, the combinatorial approach of decitabine, low dose chemotherapy drugs and G-CSF is reasonable and might be an effective strategy for pediatric MDS before transplantation.

Several limitations about our study should be considered. Firstly, the fundamental limitation is that this analysis did not include patients who received chemotherapy and/or DNA-hypomethylating therapy and did not progress to transplantation. The excellent overall responses to decitabine-concomitant treatment may not be accurately attainable for each individual among the heterogeneous MDS population. Secondly, details including chemotherapy regimens, donor types and conditioning regimens vary widely, and the robustness of the results may be impaired. Thirdly, this cohort included 28 patients with a median follow-up of 53.0 months, which is not adequate enough and may lead to a considerable bias. Finally, our analysis has the intrinsic limitation related to the retrospective nature and comparison with limited historical controls. In 2018, we had registered a multicenter study of DAC + MMR for children with MDS or AML (ChiCTR1800015872) and we are struggling for large confirmatory and prospective studies to help us to clarify whether this approach can alter the natural history of the disease. Therefore, the results in the present study must be interpreted with caution and further evidence from future prospective studies is required.

In summary, our cohort shows that probably, about 71% of the children with MDS would achieve prolonged survival with allo-HSCT. Abnormal karyotype at diagnosis, high BM blast cell percentage before transplantation and severe aGVHD may indicate undesirable outcomes. CBT is not preferred, while haploidentical HSCT might be a feasible alternative when HLA-identical HSCT is unavailable. The bridging therapy of DAC + MMR was safe and well tolerated. It appears to be more effective than AML-type chemotherapy with higher mCR rate and better survival rate in childhood MDS. Our study may provide a novel and practical bridging approach for pediatric MDS with subsequent allo-HSCT. Due to the lack of randomized controlled trials, further prospective randomized study to explicitly determine the safety and efficacy of this approach in comparison with no decitabine (AML-type chemotherapy-combined HSCT or HSCT only) are required.

## Supplementary Information

**Additional file 1: Supplementary Table 1.** The gene mutation data for the 28 children with *de novo* MDS.

## Data Availability

The datasets used and analyzed during the current study available from the corresponding author on reasonable request.
