# Supplementary material for: The effect of decitabine-combined minimally myelosuppressive regimen bridged allo-HSCT on the outcomes of pediatric MDS from 10 years’ experience of a single center
Source: BMC Pediatr. 2022 May 27;22:312. doi: 10.1186/s12887-022-03376-1 (PMC9137053; doi:10.1186/s12887-022-03376-1)
Supplement: Supplementary file 1 — Additional file 1: Supplementary Table 1. The gene mutation data for the 28 children with de novo MDS. [file 12887_2022_3376_MOESM1_ESM.doc]

Supplementary Table 1 The gene mutation data for the 28 children with *de novo* MDS

| Patient No. | Gene mutations |
| --- | --- |
| 1 | Negative |
| 2 | ITGA2B, CASP10, FGG, G6PD, HPS6, MAN2B1, PSAP |
| 3 | Not available |
| 4 | ASXL1 |
| 5 | RUNX1, SPTA1, ADAMTS13, GPX1, HPS1, TCIRG, TFRC |
| 6 | RUNX1, ITGB3 |
| 7 | Not available |
| 8 | SF1, XIAP, DNAJC21, FANCD2, FGA, MMAB, TCN2 |
| 9 | ATM, RAD21, FANCI, GAA, LYST, MYO5A, PGM1, UQCC3, USP42, VPS13A |
| 10 | Not available |
| 11 | GATA2, JAK2, MPL, ASXL1 |
| 12 | GATA2, JAK2, CSMD1 |
| 13 | Not available |
| 14 | CBLB, NCOR2, ACVRL1, CIITA, CUBN, EGLN1, F12, FANCI, FGG, MAN2B1, MEFV, PHKB, TCN2 |
| 15 | Not available |
| 16 | Negative |
| 17 | TET2, U2AF1 |
| 18 | Not available |
| 19 | Not available |
| 20 | Not available |
| 21 | ASXL1, FANCB, PDGFRB, PTPN11, CD19, CDAN1, NCOR2, RASGRP2, SLX4 |
| 22 | Not available |
| 23 | Not available |
| 24 | SMC1A, ABCC8, BMPR1A, RPS10, RUNX1, SERPINE1, ADAMTS13, BRIP1, CPN1, F12, MCFD2, PCCA, PLG |
| 25 | CBLB, PGD, PUS1, ALS2, ATM, CD36, CPN1, IL6, RPL3L, RPS27A, UMODL1 |
| 26 | ANK1, CIITA, EPB42, LYST, WAS |
| 27 | Not available |
| 28 | Not available |
